# Supplementary material for: Contrasting effects of intracellular and extracellular human PCSK9 on inflammation, lipid alteration and cell death
Source: Commun Biol. 2024 Aug 13;7:985. doi: 10.1038/s42003-024-06674-9 (PMC11322528; doi:10.1038/s42003-024-06674-9)
Supplement: Supplementary file 2 — Supplementary Information [file 42003_2024_6674_MOESM2_ESM.pdf]

Supp.figure1

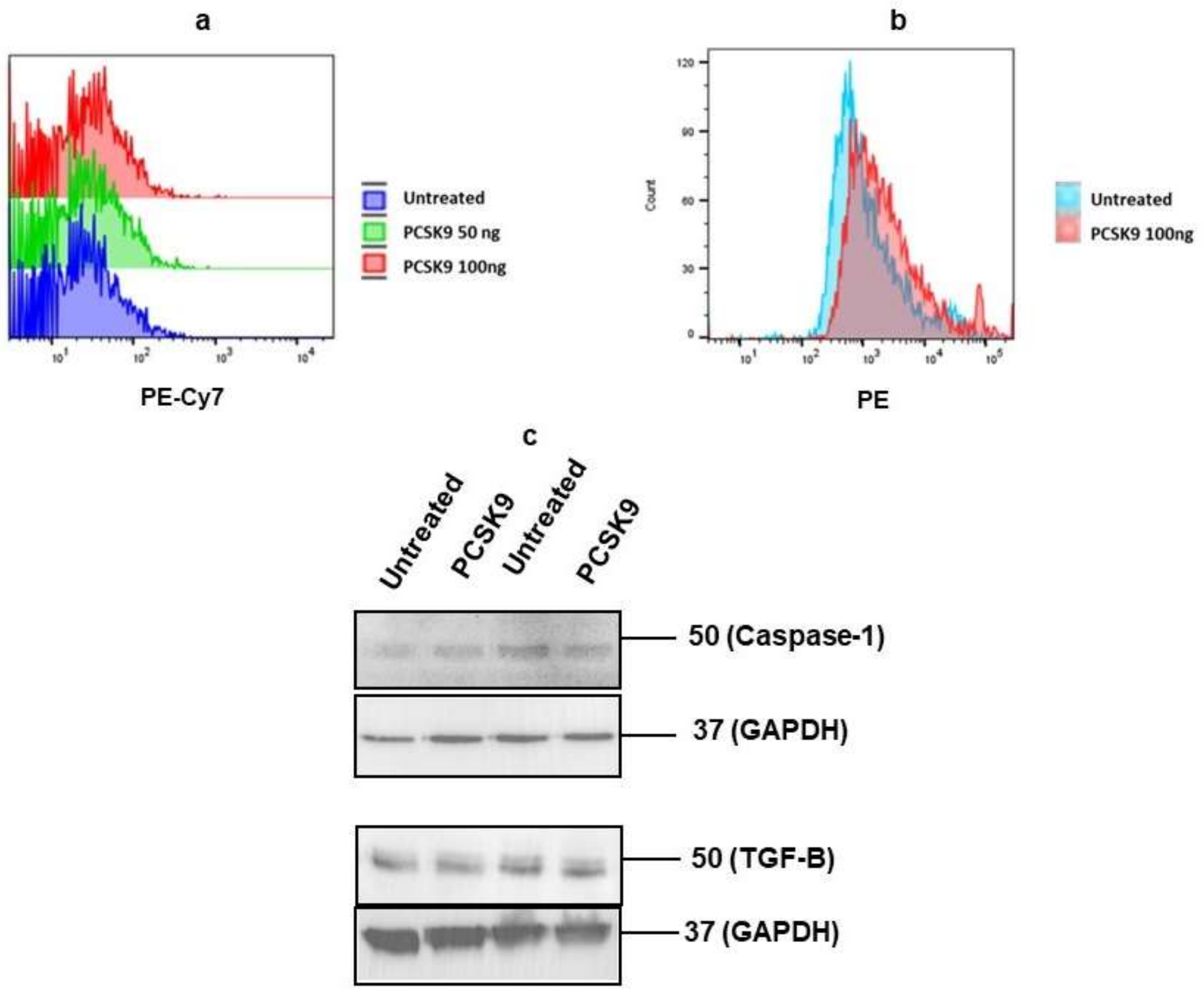


**Supp.Fig.1: Phosphorylation of NFkB and MAPKp38 and expression of caspase1 and TGF-B**.

a) PCSK9- exposed PBEC did not induce NFkB activation in PBEC. b) MAKp38 was activated in A549 cell in response to by PCSK9 exposure as evident by the shift of histogram to the right. c) Expression of caspase 1 and TGF-B in protein levels was measured from cell lysates. PCSK9 exposure did not affect expression of such in protein levels.

Supp. figure 2

a

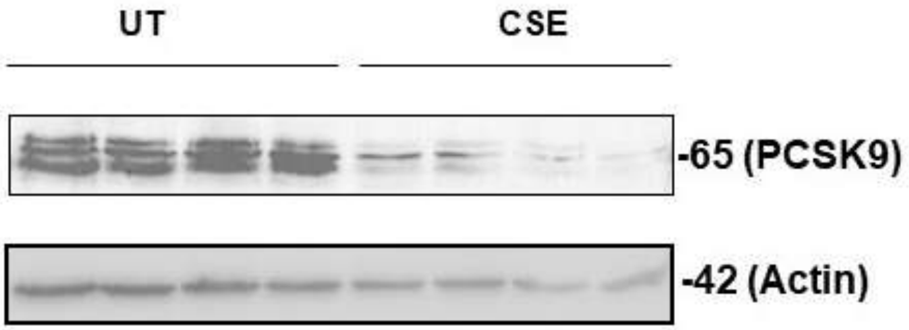


b

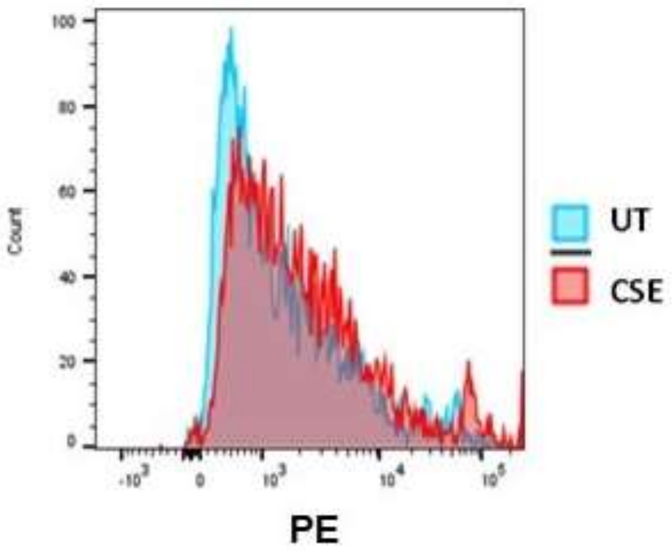


**Supp. Fig. 2: Effect of cigarette smoke extract (CSE) on cell death and PCSK9 level.**

a) CSE reduced PCSK9 in A549 cells in protein levels. b) PBEC was exposed to CSE, and death was measured by propidium iodide staining. CSE induced cell death in undifferentiated PBEC.

Supp. figure 3

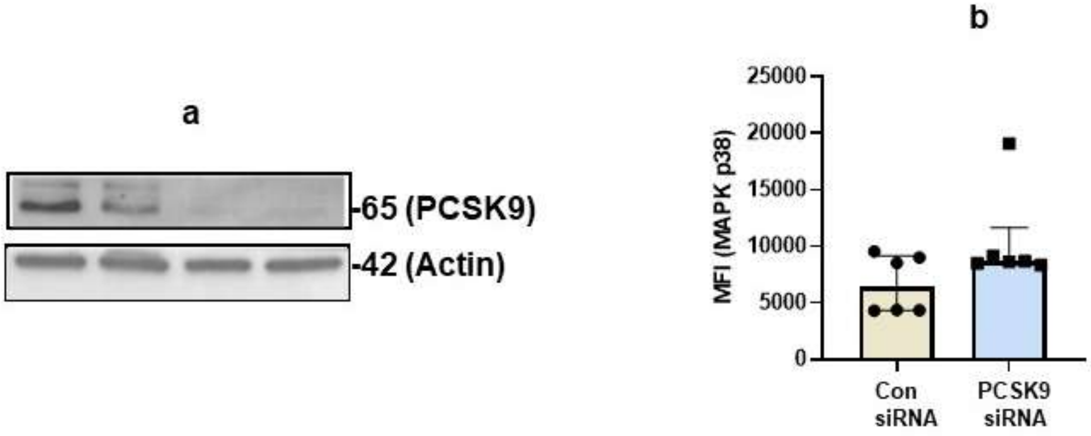


**Supp. Fig. 3: Level of PCSK9 protein level and MAPKp38 activation.**

a) PCSK9 protein level in PCSK9 siRNA transfected PBEC and control (con) siRNA transfected PBEC. B) MAPKp38 phosphorylation in PCSK9 siRNA or con siRNA transfected PBEC was measured by flow cytometry and mean fluorescent intensity was presented to represent the changes in MAPk p38 phosphorylation (n=6). Error bars represent interquartile range.

Supp. figure 4

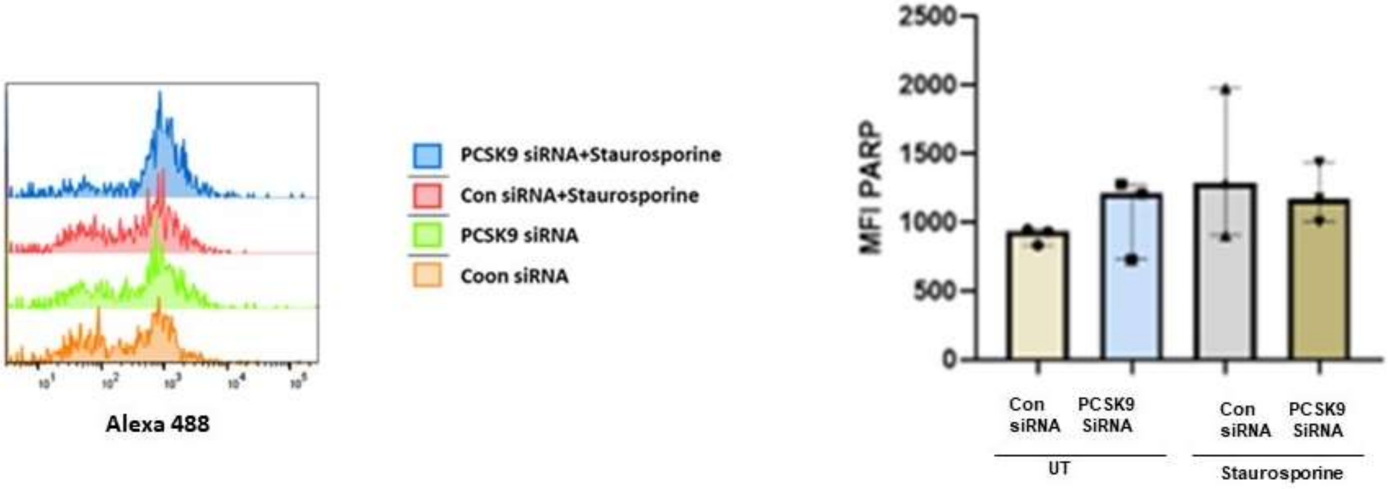


**Supp. Fig. 4: Endogenous inhibition of PCSK9 does not affect PARP activation.**

PCSK9-siRNA transfected PBEC was exposed to staurosporine. In response to the drug, PARP activation in PCSK9 or control (con) siRNA transfected PBEC in presence or absence of drugs was quantified by flow cytometry, (n=3). Error bar represent median interquartile range.

Supp. figure 5

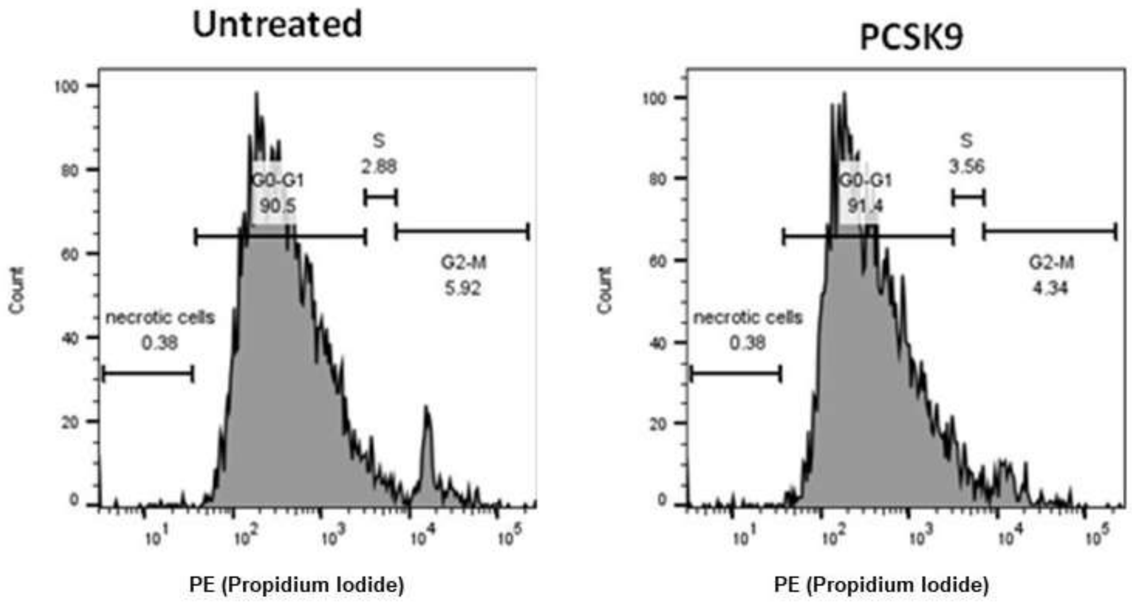


**Supp Fig. 5: Cell cycle is unaffected in response to extracellular PCSK9.**

PBEC was exposed to PCSK9 for 24 hours. Cell cycle was measured by propidium iodide staining.
