# Supplementary material for: Contrasting effects of intracellular and extracellular human PCSK9 on inflammation, lipid alteration and cell death
Source: Commun Biol. 2024 Aug 13;7:985. doi: 10.1038/s42003-024-06674-9 (PMC11322528; doi:10.1038/s42003-024-06674-9)
Supplement: Supplementary file 3 — Description of Additional Supplementary Files [file 42003_2024_6674_MOESM3_ESM.pdf]

Description of Additional Supplementary Files

File name: Supplementary data 1

Description: Western blot original images.

File name: Supplementary Data 2

Description: flow cytometry gating strategy

File name: Supplementary Data 3

Description: All the numerical values for the figures 1, 3a, 3g, 3h, 5c, 5e, 5f, 5g, supplementary figure 3b, and 4
