# Supplementary material for: Contrasting effects of intracellular and extracellular human PCSK9 on inflammation, lipid alteration and cell death
Source: Commun Biol. 2024 Aug 13;7:985. doi: 10.1038/s42003-024-06674-9 (PMC11322528; doi:10.1038/s42003-024-06674-9)

Figure 3b

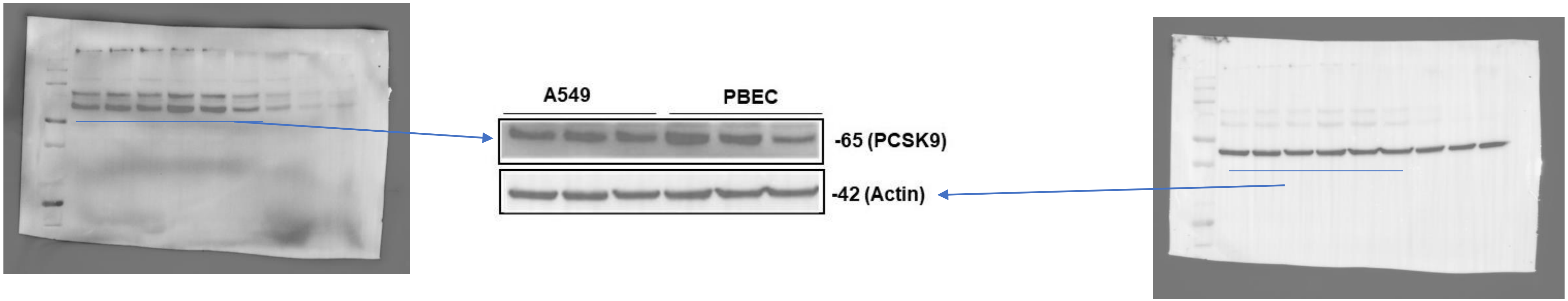

Figure: 3 D

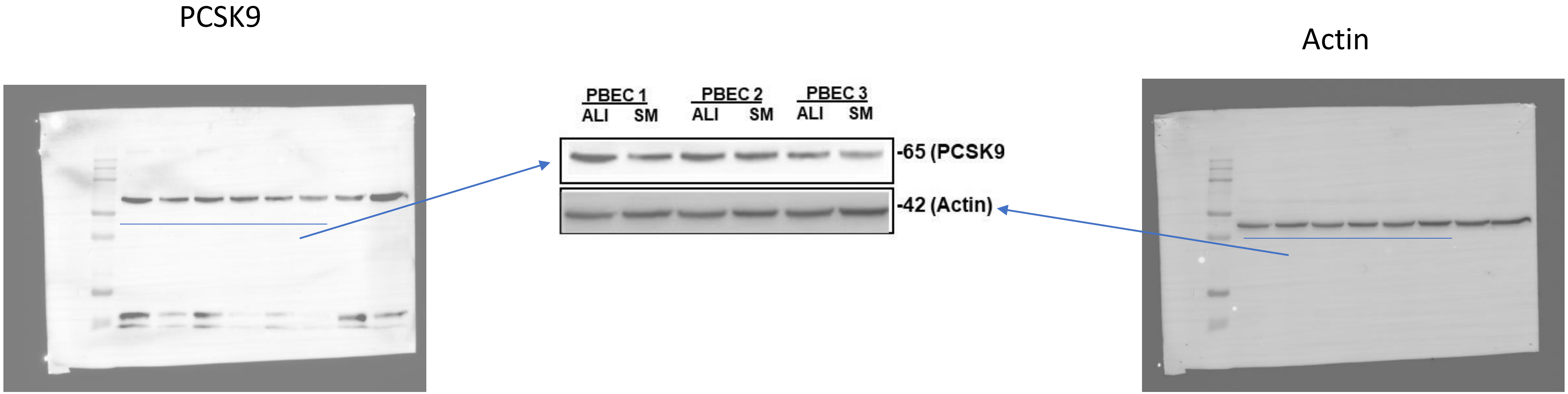
PCSK9
Actin
PBEC 1
PBEC 2
PBEC 3
ALI
SM
ALI
SM
ALI
SM
-65 (PCSK9
-42 (Actin)

Figure 3e

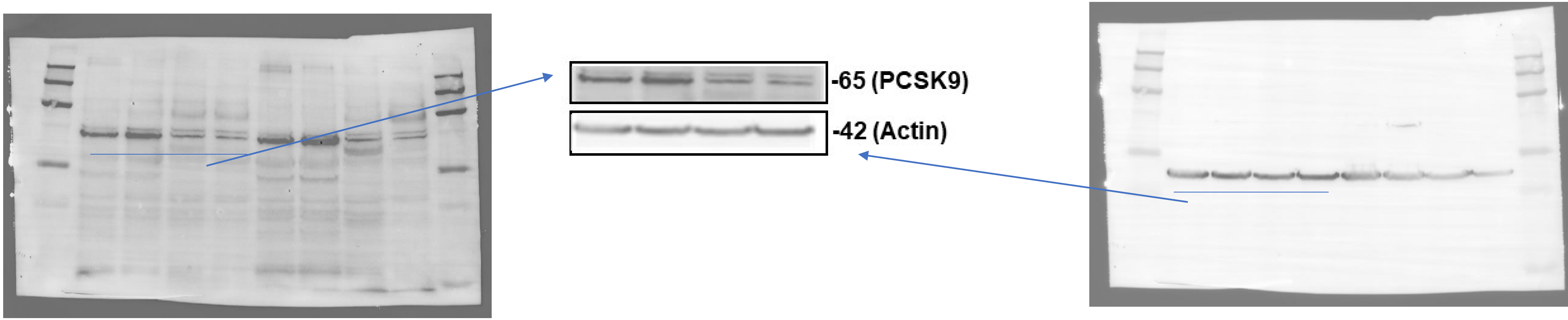

Figure 3i

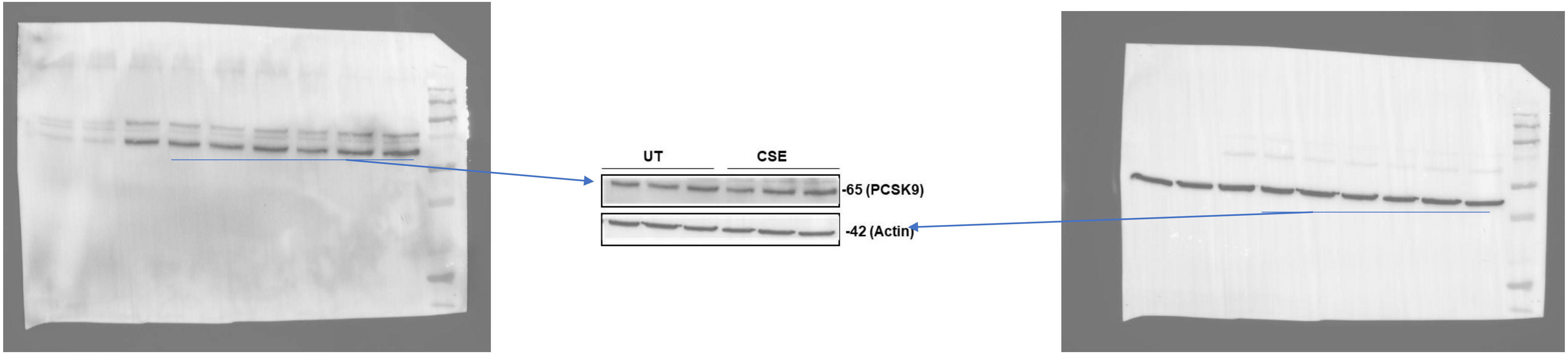

Supp figure 1c

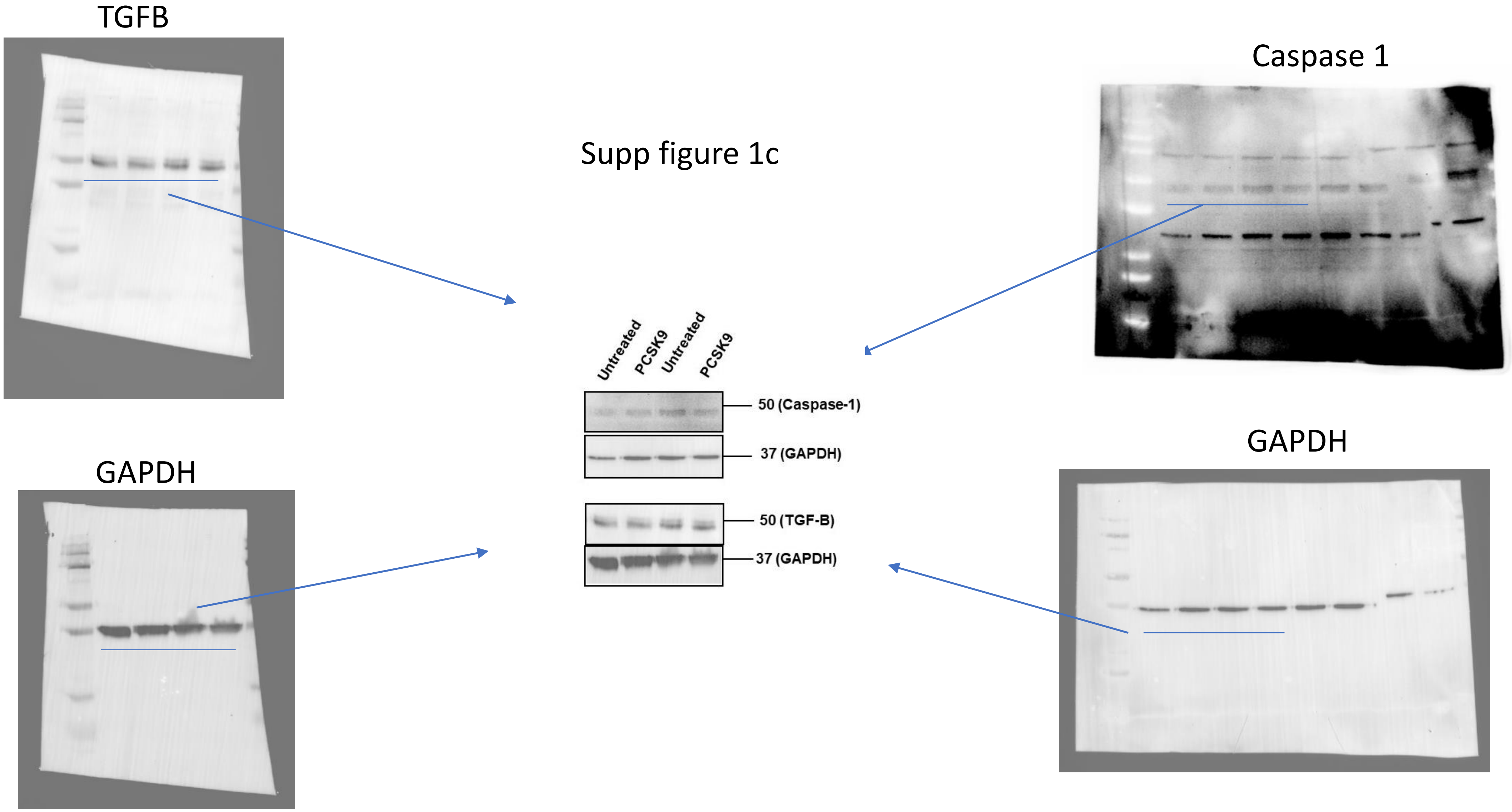

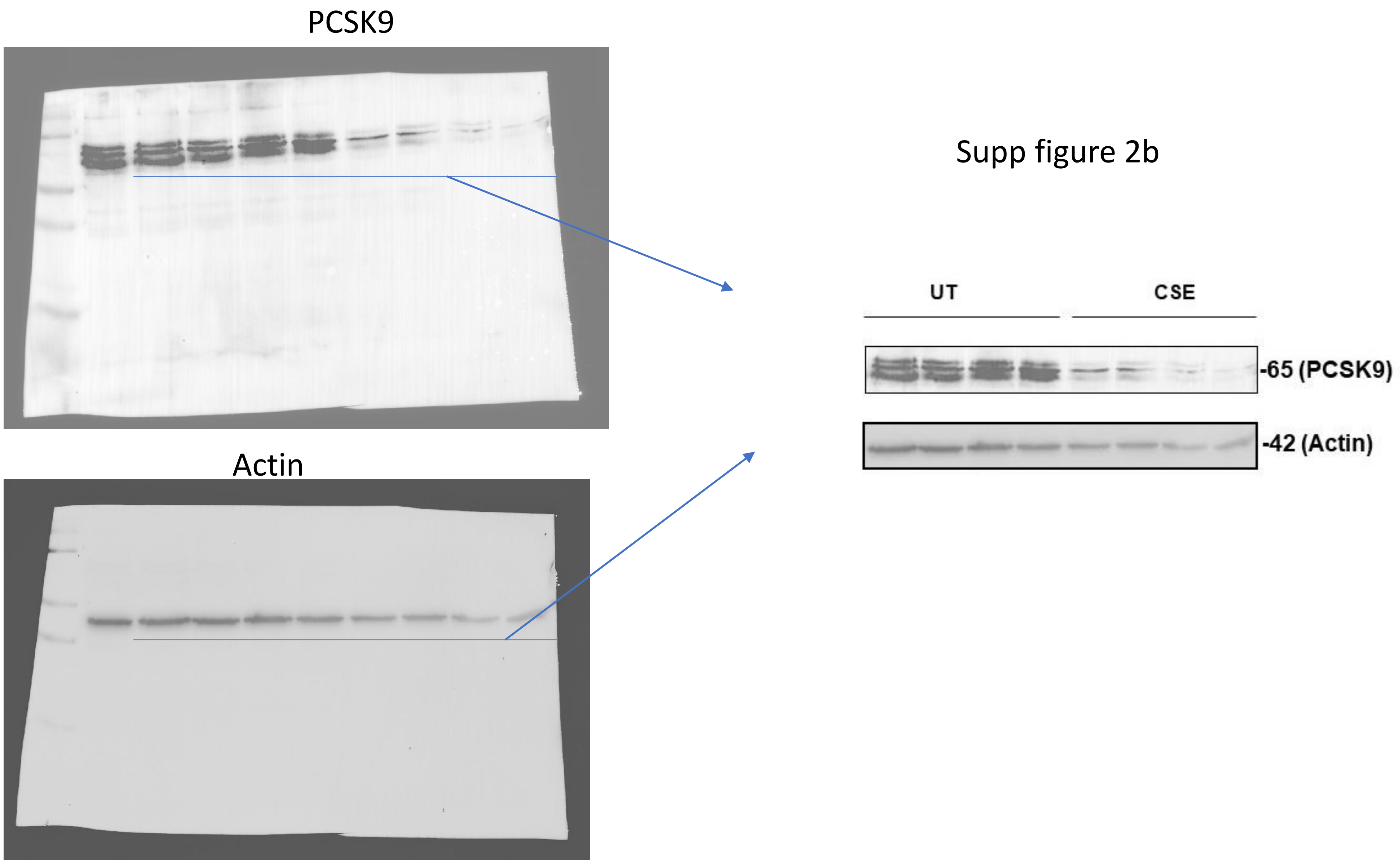
PCSK9
Actin
Supp figure 2b
UT
CSE
-65 (PCSK9)
-42 (Actin)

Supp. Figure 3a

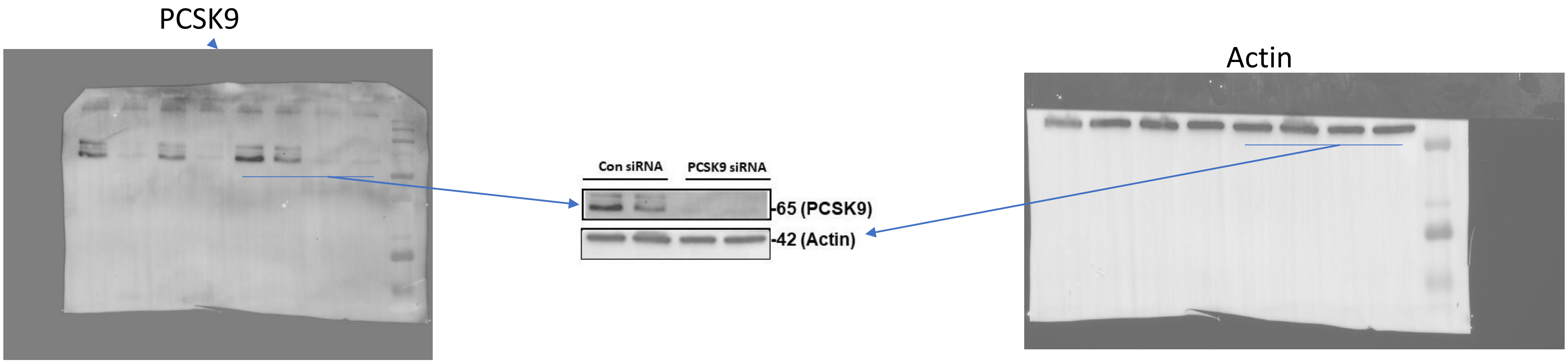

Supplement: Supplementary file 4 — Supplementary Data 1 [file 42003_2024_6674_MOESM4_ESM.pdf]
