# Supplementary material for: Contrasting effects of intracellular and extracellular human PCSK9 on inflammation, lipid alteration and cell death
Source: Commun Biol. 2024 Aug 13;7:985. doi: 10.1038/s42003-024-06674-9 (PMC11322528; doi:10.1038/s42003-024-06674-9)
Supplement: Supplementary file 5 — Supplementary Data 2 [file 42003_2024_6674_MOESM5_ESM.pdf]

Flow cytometry gating strategy for figure 5-d and supplementary figure 5.

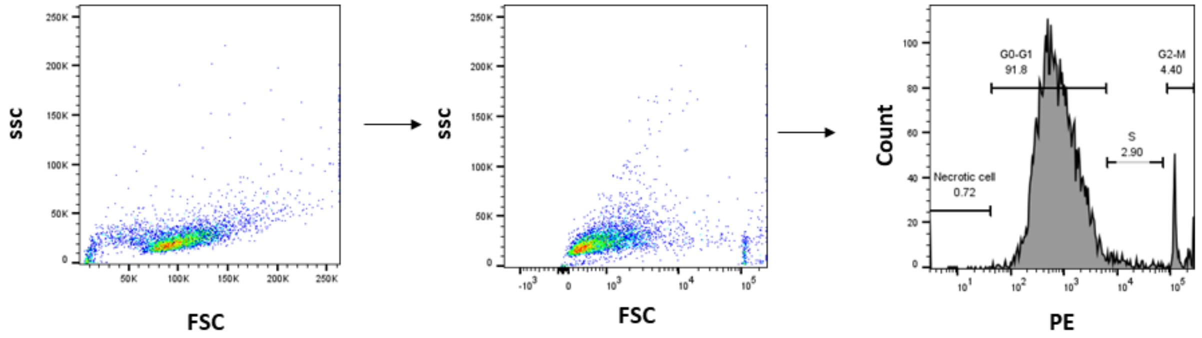

Figure: Dotplot of total cell population was converted to histogram. Necrotic or cell debris was gated in left and G0-G1, S and G2-M phase were gated. Based on gate for control condition, gate for teatment or PCSK9-suppressed condition was placed.

Flow cytometry gating strategy for figure 5-f.

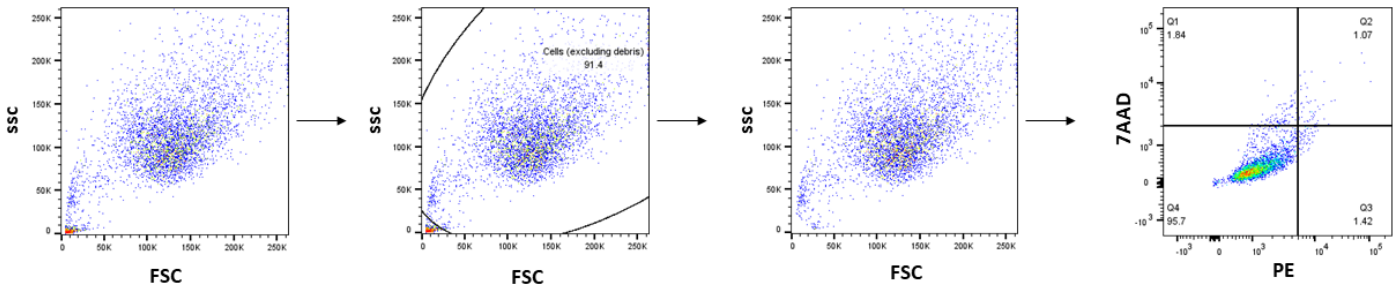

Figure: Debris was excluded from the total events and gates for 7AAD and PE (annexin V) were generated in control condition. Following the same postion, gates were generated for treatment condition and percentage of population was calculated.
